# Evaluation of Thyroid Function and Its Relation to Glycemic Status in Pregnant Women With Gestational Diabetes Mellitus

**DOI:** 10.7759/cureus.72339

**Published:** 2024-10-24

**Authors:** Vishal Vinod, Reeta Rajagambeeram, Rupal Samal

**Affiliations:** 1 Department of Biochemistry, Mahatma Gandhi Medical College and Research Institute, Sri Balaji Vidyapeeth (SBV), Puducherry, IND; 2 Department of Obstetrics and Gynaecology, Mahatma Gandhi Medical College and Research Institute, Sri Balaji Vidyapeeth (SBV), Puducherry, IND

**Keywords:** ft3, ft4, gestational diabetes mellitus, glycemic status, thyroid function, tsh

## Abstract

Background

Gestational diabetes mellitus (GDM) is a prevalent complication during pregnancy that can lead to adverse outcomes for both the mother and the fetus. It also increases the likelihood of developing type 2 diabetes mellitus (T2DM) later in life. Thyroid hormones play an essential role in regulating growth and metabolism and often coexist with diabetes mellitus (DM), affecting glucose metabolism. Pregnant women with GDM frequently exhibit thyroid issues, impacting insulin secretion and beta-cell function.

Aim

This study aims to assess thyroid function and glycemic status in pregnant women with and without GDM and to evaluate the correlation between thyroid function and glycemic status in pregnant women with GDM.

Methods

This prospective case-control study was conducted over two months at a tertiary care hospital in Puducherry, India. It included 60 cases (pregnant women with GDM, blood glucose > 140 mg/dL per DIPSI guidelines) and 60 age- and parity-matched controls. Blood samples were collected, centrifuged, and analyzed for blood glucose and serum thyroid levels (FT3, FT4, thyroid-stimulating hormone (TSH)) using the Cobas e-411 autoanalyzer through an electrochemiluminescence assay.

Results

Serum plasma glucose levels were significantly higher in cases (159.25 ± 16.22 mg/dL) compared to controls (101.6 ± 17.30 mg/dL) (p < 0.05). FT3 levels were higher in cases (3.98 ± 4.18) compared to controls (2.87 ± 0.54) (p = 0.04). The FT3/FT4 ratio was also higher in cases (3.99 ± 4.927) than in controls (2.70 ± 0.58) (p = 0.04). No significant differences were found in FT4 or TSH levels between the groups. Correlation analysis revealed no significant correlations between plasma glucose levels and thyroid function parameters.

Conclusion

Pregnant women with GDM showed significantly higher plasma glucose levels, FT3 levels, and FT3/FT4 ratio compared to normal pregnant women. These findings suggest an association between altered thyroid function, particularly higher FT3 levels and the FT3/FT4 ratio, and GDM.

## Introduction

Gestational diabetes mellitus (GDM) is caused by dysfunctional glucose metabolism in pregnant women and affects 3-10% of pregnancies. It is a transient form of glucose intolerance brought on by pregnancy-related pancreatic beta-cell dysfunction and insulin resistance, first identified during pregnancy [1].

GDM significantly impacts both maternal and fetal health [2]. Birth complications are more likely to occur in mothers with GDM [3,4]. This could result in a seven-fold increase in the chance of developing type 2 diabetes mellitus (T2DM) later in life [5].

The prevalence of GDM in the Indian population is much higher compared to other Asian countries [6,7]. GDM affects almost five million women annually in India. Current literature estimates that six million births in India alone are affected by pre-diabetes and diabetes, with GDM accounting for 90% of these cases [8].

GDM and thyroid diseases are two common endocrine disorders observed during pregnancy, with a prevalence rate of 16.6% [9,10]. The adverse outcomes of thyroid disorders in pregnancy are similar to those of GDM. Thyroid hormones play a critical role in glucose homeostasis [11]. Maternal thyroid hormones may affect blood sugar levels by regulating hepatic gluconeogenesis, intestinal glucose absorption, and glucose uptake in peripheral tissues, and also regulate mRNA and protein expression levels of glucose transporters, and alter circulating insulin levels [12]. This is also associated with effects brought about by placental lactogen, estrogen, and thyroid-binding globulin; insufficient adaptation to these changes may cause thyroid dysfunction [13].

Diabetes and thyroid disease are two common endocrine disorders observed in the adult population, with insulin and thyroid hormones being closely involved in cell metabolism. Hence, an excess or deficit of any of these hormones may result in functional derangement of the other. In euthyroid individuals with diabetes mellitus, the serum T3 levels, basal thyroid-stimulating hormone (TSH) levels, and TSH response to thyrotropin-releasing hormone (TRH) may all be influenced by glycemic status [14].

Hence, this study was planned to evaluate the thyroid status in normal pregnant women and pregnant women with GDM, and to correlate it with their glycemic status.

## Materials and methods

This prospective case-control study was conducted in the Department of Biochemistry in collaboration with the Department of Obstetrics and Gynecology at Mahatma Gandhi Medical College and Research Institute in Puducherry. The study was conducted over a period of two months after obtaining approval from the Institute Human Research Committee (IHRC) and the Institute Human Ethics Committee (IHEC) of Mahatma Gandhi Medical College and Research Institute (MGMCRI), with approval number MGMCRI/2023/03/IHEC/107. Written informed consent was obtained from the participants, and a participant information sheet was provided before data collection. Pregnant women attending the OPD and inpatient department (IPD) of the Obstetrics and Gynecology department were enrolled in the study. Participants were informed of their right to withdraw from the study at any point during the interview.

Study population

The study included two groups: Group 1 - Cases (N=60) and Group 2 - Controls (N=60). Pregnant women were sequentially recruited based on the Diabetes in Pregnancy Study group of India (DIPSI) criteria for GDM. No blinding was done in this study. It was a sequential sampling where all pregnant women who met the DIPSI criteria for GDM were recruited as cases, while normal pregnant women were recruited as controls.

Inclusion criteria

The study included pregnant women between the ages of 25 to 40 years, attending the Obstetrics and Gynecology department (OPD and IPD) during 24 to 28 weeks of gestation. Only women in their second trimester (24-28 weeks) were included. Gestational age was confirmed using the last menstrual period (LMP) and corroborated by first-trimester ultrasonography reports. Women with blood glucose levels greater than 140 mg/dL based on DIPSI guidelines for GDM were selected as cases. For controls, normal pregnant women without GDM or any other co-existing pregnancy disorders, within the same age range and gestational period, were included.

Exclusion criteria

Women with known pre-existing diabetes mellitus or under treatment with metformin; known chronic infections like hepatitis, HIV, or chronic kidney, liver, or heart disease; maternal history of hypertensive diseases in a previous pregnancy and now under prophylactic acetylsalicylate treatment were excluded.

Sample size

The sample size was 60 for cases and an equal number of age and parity-matched controls, based on a review of the literature [15]. The study assumed an alpha of 0.05 and a precision of 15% of the mean. The subjects were selected by convenient sampling based on the inclusion criteria.

Study parameters

After obtaining written informed consent from the study participants, 3 ml of venous blood was collected under aseptic precautions in a serum tube. Subsequently, the blood was centrifuged at 3000 rpm. The resulting serum was then utilized for analyzing plasma blood glucose levels and thyroid hormone levels (FT3, FT4, TSH).

Biochemical parameters were estimated based on methods established by the International Federation of Clinical Chemistry and Laboratory Medicine (IFCC). All assays were carried out on a fully automated chemistry analyzer; specifically, the blood glucose levels were examined using the glucose oxidase-peroxidase method. Additionally, serum thyroid levels, including FT3 (pg/mL), FT4 (ng/dL), and TSH (µIU/mL), were analyzed using the Cobas e-411 autoanalyzer through an Electrochemiluminescence assay. The reference ranges for thyroid function tests during the second trimester of pregnancy were TSH: 0.42-2.84 µIU/mL, FT4: 0.59-1.21 ng/dL, and FT3: 1.81-3.81 pg/mL [16]. Internal quality control was facilitated using Bio-Rad (USA) samples. External quality assessment was carried out with the help of the clinical biochemistry laboratory at CMC Hospital Vellore. Based on the glucose values obtained from DIPSI criteria, the participants were classified into cases and controls.

The DIPSI criteria [17] state that a two-hour venous blood sample of 140 mg/dL (7.8 mmol/L) in the non-fasting oral glucose tolerance tests (OGTT) is required for the diagnosis of GDM. DIPSI is a one-step process with a single glycemic value and is a modified version of WHO standards. A pregnant woman in the antenatal clinic receives a 75 g oral glucose load after undergoing a preliminary clinical examination, regardless of her fasting status or the timing of her last meal. The glucose, dissolved in 300 mL of water, is administered as soon as possible per DIPSI, and the timing is recorded. After two hours, venous blood samples are taken, centrifuged, and then analyzed.

Statistical analysis

All data are expressed as mean ± SD. An independent Student's t-test was used to find the difference between cases and controls. A p-value < 0.05 was considered statistically significant for all statistical tests. Pearson’s correlation analysis was performed to determine the correlation between blood glucose and thyroid hormones (FT3, FT4, and TSH). Data were processed using the SPSS statistical package version 20.0, IBM Corporation Software Group, USA.

## Results

All data are expressed as mean ± SD. An independent Student's t-test was used to find the difference between cases and controls. A p-value of <0.05 was considered statistically significant for all statistical tests. Data were entered and tabulated in Microsoft Excel and processed using the SPSS statistical package, version 20.0, IBM Corporation Software Group, USA.

As shown in Table 1, the independent Student's t-test showed a statistically significant difference in plasma glucose, FT3, and FT3/FT4 ratio between cases and controls, with a p-value of <0.05. However, FT4 and TSH showed no significant difference.

**Table 1:** Comparison of study parameters between cases and controls. FT3: Free Triiodothyronine; FT4: Free Thyroxine; TSH: Thyroid-Stimulating Hormone. An Independent Student's t-test was used to compare study parameters between cases and controls. *Indicates that the p-value is <0.05, which is considered statistically significant.

Parameters	Cases (n=60) (Mean ± SD)	Controls (n=60) (Mean ± SD)	t-value	P-value
Plasma Glucose (mg/dl)	159.25 ± 16.22	101.6 ± 17.30	18.828	0.00*
FT3 (pg/mL)	3.98 ± 4.18	2.87 ± 0.54	2.076	0.04*
FT4 (ng/dl)	1.30 ± 1.37	1.07 ± 0.16	2.035	0.21
TSH (µIU/ml)	2.19 ± 1.27	2.27 ± 1.39	-0.355	0.72
FT3/FT4 Ratio	3.99 ± 4.927	2.70 ± 0.58	2.005	0.04*

In Table 2, Pearson’s correlation was used to associate plasma glucose (DIPSI) with other parameters. Correlation analysis was performed to investigate the relationship between plasma glucose and thyroid function parameters, including FT3, FT4, TSH, and the FT3/FT4 ratio. The results showed no significant correlations between plasma glucose and other parameters.

**Table 2:** Correlation among plasma glucose (DIPSI) and other parameters. FT3: Free Triiodothyronine; FT4: Free Thyroxine; TSH: Thyroid-Stimulating Hormone. Pearson's correlation was used to assess the relationship between thyroid function and glycemic parameters.

Parameters	‘r’ value	P-value
Plasma Glucose vs FT3	0.128	0.163
Plasma Glucose vs FT4	0.059	0.520
Plasma Glucose vs TSH	0.013	0.886
Plasma Glucose vs FT3/FT4 ratio	0.128	0.165

## Discussion

Our findings showed a significant difference in FT3 levels between the two groups. The case group (pregnant women with GDM) had higher FT3 levels compared to the control group, indicating a statistically significant difference. This suggests an association between GDM and altered FT3 levels. Our study's findings are consistent with previous research. Rawal S et al. [18] conducted a study on thyroid markers and their association with the risk of GDM, finding that higher levels of FT3 and the FT3/FT4 ratio measured early in pregnancy were independent risk factors for GDM. FT3, the biologically active thyroid hormone, is primarily responsible for stimulating endogenous glucose production [19]. Notably, approximately 80% of circulating T3 originates from the mono-deiodination of T4, a process aided by peripheral deiodinase activity [20,21]. Thyroid hormones also regulate hepatic gluconeogenesis, glucose absorption in the intestines, and uptake in peripheral tissues, and regulate the expression of glucose transporters at both the mRNA and protein levels. They promote pathways that accelerate glycogenolysis and modify circulating levels of insulin and counterregulatory hormones [22]. Wu JN et al. [23] conducted a recent study in 2023, showing similar results to ours, with statistically significant FT3 levels (p < 0.05).

In addition to the significant difference in FT3 levels, our study also found a notable increase in the FT3/FT4 ratio in the case group compared to the control group, indicating a statistically significant difference. This finding is consistent with recent research by Raets L et al. [24], who reported that both FT3 levels and the FT3/FT4 ratio in pregnancy were significantly different and positively associated with GDM, and that FT4 levels were negatively correlated with GDM.

The ratio of serum-FT3 to FT4 is assumed to reflect the extent of peripheral conversion of thyroxine (T4) to triiodothyronine (T3). This ratio is typically stable in healthy adults but can be influenced by various factors. Deviations from the normal range of the FT3/FT4 ratio may have implications for the peripheral effects of thyroid hormones [25]. An altered FT3/FT4 ratio, either elevated or reduced, has been associated with metabolic disorders affecting lipid profiles, blood pressure, and insulin resistance. The decrease in FT4 levels is thought to be due to increased peripheral deiodinase activity, leading to enhanced conversion of FT4 to the biologically active FT3, thereby increasing the FT3/FT4 ratio. Wang Y et al. found a positive association between the FT3/FT4 ratio in early pregnancy and the risk of GDM [26].

When comparing FT4 and TSH values between the cases and controls, no statistically significant difference was found. Contrary to our study, Huang K et al. [27] and Raets L et al. [24] found in their studies that women with GDM have higher levels of TSH. The differing results could be ascribed to several factors, such as variations in the demographic composition of the study populations, including differences in ethnicity or genetic background, and the use of different diagnostic criteria for GDM which might influence the association between TSH levels and GDM.

No significant correlation was found between plasma glucose and other parameters. Consistent with our findings, Özışık H et al. [28] also reported no significant differences in TSH levels and FT4 concentrations between GDM and non-GDM groups. They observed a significant negative correlation between TSH and FT4 in the GDM group, suggesting potential dysregulation in thyroid function among women with GDM.

In this study, several strengths contribute to the reliability and validity of our findings. The prospective case-control design offers a comprehensive analysis of the association between FT3 levels, the FT3/FT4 ratio, and GDM. The use of appropriate statistical tests and the consideration of a p-value <0.05 as statistically significant strengthen the validity of our findings. Our findings support our hypothesis that GDM is associated with altered thyroid functions.

Limitations

Our study has several limitations that should be acknowledged. We did not account for potential confounders such as BMI and HbA1c values, which could influence the results, and we lacked access to anti-thyroid peroxidase (anti-TPO) and anti-thyroid antibody values, which could have provided additional insights into thyroid function. The relatively small sample size, due to the short-term nature of the project, also limits our ability to draw broader conclusions. These limitations suggest that while our results point to a potential link between thyroid hormones and GDM, further research with larger, more comprehensive studies is necessary to confirm and strengthen our conclusions.

Implications

Our findings suggest that increased FT3 levels and the FT3/FT4 ratio in early pregnancy may be linked to a higher risk of GDM. Clinically, this emphasizes the importance of monitoring thyroid function in pregnant women, as early detection of altered hormone levels could offer opportunities for intervention, potentially improving outcomes for both mothers and infants.

Future studies should focus on confirming these findings with larger sample sizes and more comprehensive data, including confounders such as BMI, HbA1c, and thyroid antibodies. Investigating whether treating thyroid dysfunctions, such as hyperthyroidism, reduces the risk of GDM would also be valuable. Additionally, the FT3/FT4 ratio could be explored as a potential biomarker for predicting GDM, further enhancing early detection and prevention strategies.

## Conclusions

In our study, we found significant differences in plasma glucose levels, FT3 levels, and the FT3/FT4 ratio between pregnant women with GDM and those without (controls). Specifically, plasma glucose, FT3 levels, and the FT3/FT4 ratio were higher in the GDM group, contributing to increased blood glucose levels due to synergistic effects with insulin resistance. Additionally, we observed no significant correlation between GDM and plasma-free thyroxine (FT4) levels, indicating similar FT4 levels in both groups. Furthermore, there was no significant association between GDM and plasma TSH levels, suggesting that TSH levels did not differ between the GDM and non-GDM groups in our study population.
